# Paternal Postpartum Bonding and Its Predictors in the Early Postpartum Period: Cross-Sectional Study in a Polish Cohort

**DOI:** 10.3389/fpsyg.2021.628650

**Published:** 2021-04-09

**Authors:** Łucja Bieleninik, Karolina Lutkiewicz, Paweł Jurek, Mariola Bidzan

**Affiliations:** ^1^Department of Clinical and Health Psychology, Faculty of Social Sciences, Institute of Psychology, University of Gdańsk, Gdańsk, Poland; ^2^GAMUT-The Grieg Academy Music Therapy Research Centre, NORCE Norwegian Research Centre, Bergen, Norway; ^3^Department of Social Psychology, Faculty of Social Sciences, Institute of Psychology, University of Gdańsk, Gdańsk, Poland

**Keywords:** bonding, anxiety, stress, postpartum depression, parenting (MeSH), newborns, fatherhood and building early relationships

## Abstract

**Introduction**: Parental postpartum bonding has been studied by many researchers focusing on maternal bonding. The objective of this study was to examine the psychological and socio-demographic predictors of paternal postpartum bonding in the early postpartum period.

**Methods**: In this cross-sectional study, 131 couples (fathers median age of 32.37 years, *SD* = 4.59; mothers median age of 30.23 years, *SD* = 3.90) of newborns from full-term pregnancies were recruited from November 2019 until March 2020. The primary outcome was paternal postpartum bonding as measured by the Postpartum Bonding Questionnaire (PBQ). Secondary outcomes included: maternal and paternal anxiety [with the Generalized Anxiety Disorder (GAD) Assessment]; maternal and paternal stress [with the Parental Stress Scale (PSS)]; maternal depressive symptoms [with the Edinburgh Postpartum Depression Scale (EPDS)]; and maternal and paternal socio-demographic variables as fathers’ presence at childbirth, education level, age, and parental experience.

**Results**: Paternal postpartum bonding was significantly correlated with paternal anxiety (moderate strength), maternal stress (strong correlation), and maternal postpartum bonding. No significant correlations between paternal postpartum bonding, maternal depression symptoms, and maternal anxiety were found. The mediating role of paternal stress in paternal postpartum bonding was proven. Paternal anxiety strengthens paternal stress (*b* = 0.98). Further, a high level of paternal stress disrupts paternal postpartum bonding (*b* = 0.41). Results of regression analyses have revelated that maternal infant bonding (*p* < 0.01) and paternal stress (*p* < 0.01) are the only predictors of parental postpartum bonding across all included variables. None of investigated socio-demographic variables were associated with paternal postpartum bonding.

**Conclusion**: Notwithstanding limitations, the current findings add to a growing body of literature on paternal postpartum bonding. The results have shown that paternal mental health is related to parental postpartum bonding directly after delivery.

**Clinical Trial Registration:** ClinicalTrials.gov Identifier: NCT04118751.

## Introduction

Many researchers have examined parental bonding over the last few decades, since the concept was initially described in the 1970s (Klaus and Kennell, 1970, 1976; Klaus et al., 1972). The process of forming a healthy bond between parents and their newborn baby in the early postpartum period is important due to its long-lasting impact on the future parent – infant relationship (Mihelic et al., 2017; Nelson et al., 2019), the child’s survival and consequently the child’s development (Leckman et al., 2004; Nakano et al., 2019). Here, “maternal – infant bonding” (MIB) is defined as the emotional tie of a mother to her baby, as it gradually unfolds in the first year of a child’s life’ (Kinsey and Hupcey, 2013, p.7). Bonding development may look differently for mothers and fathers. A paternal – infant bond is often defined as the relationship between a father and his child. It starts to emerge early in pregnancy and further develops becoming more prevalent 2 months after childbirth (Anderson, 1996).

Based on existing knowledge, the postpartum period is considered the most difficult period for first-time fathers (Chin et al., 2011) due to new tasks, new emotions, new role development, and a lack of father-specific resources (Kumar et al., 2018). Society’s expectations of a father’s contribution in parenting have increased over time (Opondo et al., 2016). The evolution of the paternal role and a lack of father-specific models may make it more challenging to fulfill all of the modern expectations and have a proper understanding of how to be a competent father (Barclay and Lupton, 1999). Thus, first-time fathers may find it challenging to adjust to their new role after childbirth and live up to these new demands. Even in the case of a healthy newborn from full-term pregnancies, they can feel frustrated and uncertain, by not having enough opportunities to strengthen the emotional tie with their baby (Barclay and Lupton, 1999; Premberg et al., 2008), because of the mother’s primary-caregiver role. What is more, personal and family changes may increase vulnerability to psychological stress in the early post-partum period (Epifanio et al., 2015). First-time fathers may feel less confident than new mothers with a baby, and may feel excluded in the close relationship that is growing between the mother and her baby. As a consequence, fathers may experience an increased level of stress and anxiety and/or depression, which later can be a risk factor of an impaired paternal bond with the baby.

A father’s mental health during the antenatal and postpartum periods is a relatively under-researched topic in comparison with maternal mental health at that time (Ilska and Przybyła-Basista, 2014; Kucharska, 2020; Lutkiewicz et al., 2020). The prevalence of perinatal depression among fathers has been shown to be about 10% at the very beginning of pregnancy increasing to 25% around 6 months after birth (Paulson and Bazemore, 2010). Other research showed that paternal postpartum depression during the first year after childbirth can range between 4 and 25% (Soliday et al., 1999; Kim and Swain, 2007). This prevalence might be underreported due to the use of different measures, various cut-off scores, different measuring time-periods, and various other social and cultural factors. Factors for paternal postpartum depression include negative emotions, financial concerns/instability, balancing work–life demands, older age, low education levels, marital problems (Kumar et al., 2018), and anxiety (Chin et al., 2011; Kowlessar et al., 2015). Some studies claimed that a father’s mood and emotional well – being are associated with their partner’s mood (Deater-Deckard, 1998; Nishimura and Ohashi, 2010) and that these can influence each other (Paulson and Bazemore, 2010; Vismara et al., 2016). Research has also shown that paternal depression is significantly connected to maternal depression (Pinheiro et al., 2006; Letourneau et al., 2011; Edward et al., 2015; Nishimura et al., 2015).

Parental stress is a bio-psycho-social construct, where demands are placed so high that a parent does not have the available resources to fulfill them (Abidin, 1995). It may be experienced as a feeling of being overwhelmed, uncertainty in the parental role, or a feeling of being unsatisfied (Pasley et al., 2002). Anxiety among fathers also increases as pregnancies progress, and comes to a peak 2 months after birth impacting 11.6–14.2% of fathers (Koh et al., 2015). Research has shown that an increased level of anxiety is related to a decrease in paternal competence (Mercer and Ferketich, 1994). With the evolution of a father’s role and societal demands, paternal stress has become more of a common experience in fatherhood (Pasley et al., 2002). Examining the experience of fathers’ stress is an area that is overlooked in research. Knowing more about a father’s experience of anxiety is important in enhancing parenting self-efficacy and achieving a more satisfying and successful transition to fatherhood. It is necessary to analyze paternal stress after childbirth as stress and anxiety are significant risk factors for depression in men (Vismara et al., 2016). On the other hand, anxiety together with stress and depression among fathers can also be interdependent (Vismara et al., 2016). A study conducted by Wee et al. (2015) among fathers during pregnancy found that high levels of anxiety in early pregnancy is related to high levels of depression and stress in late pregnancy.

Men also go through a process of transition to fatherhood and that the postpartum period is especially a vulnerable time for fathers (Koh et al., 2015). The paternal mental states as well as paternal bonding are crucial during pregnancy and after childbirth as they are connected with the developmental outcomes for children (Ramchandani et al., 2013). For example, parents with depression have a tendency to experience an increase in negative emotions and helplessness, which can translate into a lower quality of interactions with the baby (Kim and Swain, 2007). Findings suggested that a stronger paternal bond is associated with better infant outcomes (Ramchandani et al., 2013). For example, Ramchandani et al. (2013) found that disengagement and a poor connection between fathers and their babies as early as the third month of a baby’s life, predict early negative behavioral outcomes in children at the age of one. Low paternal engagement is correlated with higher infant mortality and the poorer well – being of children (Geary, 2000). Thus, health-care professionals should appropriately support first-time fathers in the transition to fatherhood as a better paternal mental state increases the chance for a more satisfying bond with a child.

Parental postpartum bonding has been studied by many researchers focusing on maternal bonding. However, how fathers’ bond with their child and what predictors influence this process is only begging to emerge in research. This study aims to contribute to this growing area of research by exploring the experience of paternal bonding with a newborn child. The purpose of this study was to assess psychological (paternal and maternal) and socio-demographic predictors of paternal postpartum bonding. The following research questions were posed:

1. Whether paternal postpartum bonding is associated with fathers’ mental health (anxiety and stress)?
2. Whether paternal postpartum bonding is connected with mothers’ mental health (maternal depression, stress, and anxiety) and maternal postpartum bonding?
3. Whether paternal postpartum bonding is associated with being a first-time father, a father’s presence at childbirth, paternal age, and education level?

Based on the aforementioned research results, it was possible to hypothesize that fathers with a high level of stress and anxiety are more likely to experience problematic paternal postpartum bonding. It could conceivably be hypothesized that the occurrence in mothers of depression symptoms, a high level of anxiety and stress is also associated with fathers’ problematic paternal postpartum bonding. Finally, we assumed that a lack of father’s presence at childbirth, lack of experience with having a previous child (being a first-time father), older age, and low education level is associated with problematic paternal bonding in the early postpartum period.

## Materials and Methods

### Study Design

This study was designed as a cross-sectional study, which is part of a larger longitudinal study on families after birth (ClinicalTrials.gov ID: NCT04118751). The procedures of this study were approved by the Research Ethics Board at the University of Gdansk (no 7/2019, date of approval: April 29, 2019).

### Population

Enrollment was provided by trained assistants in accordance with predetermined eligibility criteria through medical records of females who gave full-term birth (defined as above 37 weeks of gestation) in the Neonatology, Gynecology, and Obstetrics Unit of the University Clinical Center in Gdansk (Poland). Couples (between the age of 18 and 50 years) were recruited for the study after a woman’s delivery and were included after providing a written consent form. Our rationale to the cut-off age limit is because age of parents may affect the bond. The advanced age of parents is related to reproductive health (e.g., risk of infertility, fetal anomalies, pregnancy loss, obstetric complications, and stillbirth), while lower paternal age is linked with the adverse mental health outcomes (e.g., depression, substance abuse, and posttraumatic stress disorder) in the perinatal period (Heath et al., 1995; Pennings, 1995; Hodgkinson et al., 2014; SmithBattle and Freed, 2016).

### Trial Procedures

In order to identify fathers, the mothers of newborns hospitalized in the postpartum ward were asked first for their willingness to participate in the project. Trained assistants contacted potential mothers within the first 24 h after giving birth in order to provide an oral explanation of the proposed research project and an informed consent form to sign. Assistants were available at the unit to answer question about the project. It was highlighted that enrolment in the study is voluntary and parents could refuse to participate without giving any reason. No participation in the project had bearing on the medical care parents and their child received at the unit. In addition, parents were informed whom to contact in case on further questions.

Data for this study were prospectively collected in the early postpartum period (1–3 days post-partum) from fathers who were asked to complete paper datasets of anonymous questionnaires regarding their subjective level of stress, anxiety, and bonding with their newborns as well as a socio-demographic questionnaire. A relevant hospital policy in Poland is that a mother with her baby stays at the hospital 2–3 days after delivery. During their stay, fathers of newborns are encouraged and welcomed to visit their children. Since the early post-partum period is a high-risk time for parents’ mental health outcomes (especially for first-time fathers), we used parental presence in post-delivery wards to gather unique data on paternal outcomes. In order to maintain confidentiality, paper datasets were secure with participant ID retrieved from their medical card. The ID was unrelated to the subjects’ identifiers with one exception – the informed consent form; however, the consent forms were stored in a place accessible only to the project manager, separated from the paper datasets.

### Measures

#### Paternal Postpartum Bonding/Maternal Postpartum Bonding

Postpartum Bonding in mothers and fathers was measured with the Postpartum Bonding Questionnaire (PBQ, Brockington et al., 2006b). This is a self-report questionnaire widely used to evaluate the occurrence of disturbances of bonding formation between a parent and child during the postpartum period. It contains 25 items evaluated on a six-point Likert scale (each item scores between 0 and 5). The sum of scores ranges from 0 to 125. Higher scores indicate problematic bonding (Brockington et al., 2001). The questionnaire is divided into four factors:

1. Factor 1: general factor (includes 12 items) with a cut-off score of 11 (higher scores indicate bonding disorder).
2. Factor 2: rejection and pathological anger (includes seven items) with a cut-off score of 16.
3. Factor 3: anxiety about the infant (includes four items) with a cut-off score of 9.
4. Factor 4: incipient abuse (includes two items) with a cut-off score of 2 (Brockington et al., 2001).

Postpartum Bonding Questionnaire was chosen because it is a world-wide instrument translated and validated into different languages, including German (Reck et al., 2006; van Bussel et al., 2010), Spanish (Garcia-Esteve et al., 2016), Chinese (Siu et al., 2010), Japanese (Kaneko and Honjo, 2014; Suetsugu et al., 2015; Ohashi et al., 2016), and Jordan (Thekrallah et al., 2019). The Cronbach *α* coefficients of reliability were calculated as 0.85 for the German version (Reck et al., 2006), 0.90 for the Spanish (Garcia-Esteve et al., 2016), and 0.720 for Jordanian mothers (Thekrallah et al., 2019). Translation to Polish was done by the investigator (ŁB) according to World Health Organization recommendations,^1^ after obtaining approval from the author of the PBQ (private correspondence from May 29, 2018 until November 27, 2018). Since there was no Polish adaptation of the PQB and at the same time there were inconsistent reports on the reliability and validity of factors 3 and 4 (Brockington et al., 2006a; Wittkowski et al., 2007), an exploratory factor analysis (EFA) was used to identify meaningful factors underlying the Polish version of the scale. Conducting EFA, we used principal component analysis with varimax rotation. Reliability was calculated using Cronbach’s alpha for all scale’s internal consistency. We reported using the score and its 95% CI. The suitability for EFA was examined by Bartlett’s test of sphericity, *χ*^2^(276) = 1897.27, *p* < 0.001, and the Kaiser-Meyer-Olkin measure of sampling adequacy (0.71). The number of factors to be retained was guided by Kaiser’s criterion (eigenvalues above 1) and consideration for the amount of variance explained by the factor solution, and that two criteria favored the four-factor structure (62% of the total variance explained using 22 items after excluding those with a factor loading less than 0.3). However, the assignment of items to factors turned out to be different compared to the original version of the tool (see Brockington et al., 2006b). Supplementary Table S1 in the Supplementary Material presents EFA standardized factor loadings based on current study data. Since the factor structure of the Polish version of the tool differs from that shown by the authors of the original version, in further analyses, we used only the overall scale score calculated as the sum of all PBQ’s items.

#### Paternal Anxiety/Maternal Anxiety

Level of anxiety in mothers and fathers was measured with the Generalized Anxiety Disorder (GAD) Assessment (GAD-7, Spitzer et al., 2006). This is a self-report scale widely used to evaluate the severity of generalized anxiety. It contains seven items evaluated at a four-point Likert scale. Responders are asked to answer the following question: “Over the last 2 weeks, how often have you been bothered by the following problems?” (not at all, several days, more than half the days, and nearly every day). The sum of scores ranges from 0 to 21 with the following cut-off points: 5 interpreted as mild levels of anxiety, 10 – moderate; and 15 – strong. GAD-7 differentiates GAD from comorbid depression. Evaluation of psychometric properties showed that GAD-7 is a reliable and valid measure to capture anxiety symptoms in the general population (Löwe et al., 2008; Hinz et al., 2017) as well as in psychiatric patients (Johnson et al., 2019). The Cronbach *α* coefficients of reliability were calculated as 0.92, while intraclass correlation as 0.83 (Spitzer et al., 2006). To our best knowledge, there has been no study undertaken in order to evaluate the psychometric properties of GAD-7 based on Polish cohort; thus, we followed norms proposed by Spitzer et al. (2006).

#### Paternal Stress/Maternal Stress

Level of stress in mothers and fathers was measured with the Parental Stress Scale (PSS, Berry and Jones, 1995). This is a self-report scale used to measure the stress level experienced by mothers, fathers, stepparents, and foster parents, as well as to measure parenting stress associated with grandparenting (Louie et al., 2017). Items represent positive (e.g., emotional benefits, personal development) and negative (demands on resources, restrictions) themes of parenthood. PSS contains 18 items evaluated at a five-point Likert scale (strongly disagree, disagree, undecided, agree, and strongly agree) with the sum of scores ranges from 18 to 90. Higher scores indicate a higher level of parental stress (Berry and Jones, 1995). The Cronbach *α* coefficients of reliability were calculated as 0.83 while inter-item correlation as 0.23 (which was in line with authors expectation; the items focus on variety of examples in terms of broad construct; Berry and Jones, 1995). The scale was translated into five languages and used in eight countries (Australia, Canada, China, India, Ireland, Spain, Malaysia, and United States; Louie et al., 2017). To our knowledge, the scale was not used for clinical research in Poland so far. Translation was done into Polish by the investigator (ŁB) according to World Health Organization recommendations.

#### Maternal Depressive Symptoms

Maternal depressive symptoms were evaluated with the Edinburgh Postpartum Depression Scale (EPDS; Cox et al., 1987; Polish translation: Bielawska-Batorowicz, 1995). This is a self-report scale used worldwide for screening depression in both women and men during antenatal and postnatal periods. The scale indicates how the parent felt during the previous week. EPDS contains 10 items evaluated at a four-point Likert scale (each question scores between 0 and 3) with the sum of scores ranging from 0 to 30. Higher scores indicate more severe depressive symptoms. Research has reported that EPDS is a reliable and valid measure for use with diverse cultural, geographical, and non-English speaking populations (Department of Health, Government of Western Australia, 2006; McBride et al., 2014). For each translated version, another cut-off score is recommended for optimal sensitivity (Department of Health, Government of Western Australia, 2006). Psychometric properties of the Polish version of the EPDS indicate that the Cronbach α coefficients of reliability were calculated as 0.91 while intraclass correlation as 0.95 with the scale’s sensitivity of 96% and the specificity was 93% for the cut-off of 13/14 (Kossakowska, 2013).

#### Other Parental Outcomes

The following socio-demographic information were collected from both parents: age, marital status, education level, and work situation; number of children in the household including a newborn participating in this study.

#### Other Children Outcomes

The following medical items of children were collected from hospital records: child gender (male/female), birth weight (grams), delivery route (vaginal/caesarean), and final APGAR score (APGAR points).

### Statistical Methods Plan

Descriptive statistics were characterized by descriptive methods [mean (SD), median (range), *n* (%)]. Data management and analysis were performed using SPSS, version 26. The statistical plan was divided into three phases.

1. Descriptive data were generated for all variables. In the first step, the Pearson product moment correlation coefficient was used to determine the relationship between paternal postpartum bonding and study variables (paternal anxiety, paternal stress, maternal depression symptoms; maternal postpartum bonding, maternal anxiety, maternal stress).
2. To test the mediating model of paternal anxiety on paternal postpartum bonding *via* paternal stress, we used Model 4 of the PROCESS macro for SPSS (Hayes, 2013). Whereas classic mediation (Baron and Kenny, 1986) has the assumption of normality of sampling distribution, this test assumes a nonparametric distribution and relies on the bootstrap procedure. We used 5,000 bootstrap resamples to generate CIs for the indirect effect of paternal anxiety on paternal postpartum bonding *via* paternal stress [effect = 0.40; Bootstrap lower level 95% CI (LLCI) = 0.21; Bootstrap upper level 95% CI (ULCI) = 0.62].
3. The third set of analyses were linked with predicators of paternal postpartum bonding evaluation. A regression analysis was calculated on the whole group of fathers to examine the relationship between paternal postpartum bonding (explained variable) and only those variables, which were significant (*p* < 0.01) in the first phase. In addition, we added the following explanatory variables: fathers’ presence at childbirth, fathers’ educational level, fathers’ age, and fathers’ parental experience (being a first-time father).

## Results

### The Characteristics of Study Group

Individuals were recruited from November 2019 to March 2020. The sample comprised 131 couples with a mean mothers’ age of 30.23 years (range 22–45, *SD* = 3.90) and mean fathers’ age of 32.37 years (range 22–50, *SD* = 4.59). Table 1 shows the socio-demographic characteristics of the sample. The large majority of the sample comprised married couples (75%), with the remaining couples living together, but not married (23%); 2% of the interviewed couples did not answer the question about their marital status.

**Table 1:** Socio-demographic characteristics of the study sample.

Demographic variable	Mothers		Fathers	
	*n*	%	*n*	%
Education level				
Primary/elementary or less	1	0.8	–	–
Secondary school, but not completed	2	1.5	6	4.6
Secondary school graduate	19	14.5	31	23.7
University/college, but not completed	5	3.8	11	8.4
University degree (Bachelor or equivalent)	22	16.8	22	16.8
University degree (Master or equivalent)	76	58.0	58	44.3
University degree (PhD or equivalent)	3	2.3	3	2.3
Missing	3	2.3	–	–
Work situation				
Full-time employed	93	71.0	91	69.5
Part-time employed	7	5.3	1	0.8
Self-employed	8	6.1	31	23.7
Student full-time	1	0.8	–	–
Student part-time	2	1.5	–	–
Stay-at-home parent	10	7.6	1	0.8
Unemployed and seeking work	3	2.3	1	0.8
Missing	7	5.4	6	4.6

All of the children were delivered on time (mean age in weeks was 39.71, *SD* = 1.16); 53% of children were vaginal delivery and 47% by caesarean section; the mean weight of the children after birth was 3469.51 g (*SD* = 450.29); 92% of children received the maximum number of points on the Apgar scale. In 28% of cases, fathers were present during childbirth. Among the children of the surveyed couples, 66 were girls and 65 boys. For 56% of couples, it was their first child.

### Correlation Between Paternal Bonding and Study Variables

Table 2 reports the means, SDs, Pearson’s zero-order intercorrelations, and reliabilities (on the diagonal) of the variables under study. The average intensity of postpartum bonding was measured in the group of fathers and mothers. Both mothers and fathers did not experience problematic postpartum bonding. It can be seen from the data in Table 2, the average level of paternal postpartum bonding is 6.63 points (range 0–33; *SD* = 6.68), while the average level of maternal postpartum bonding is 7.95 (range 0–30; *SD* = 6.64). The difference of means is statistically significant (*t* = −2.39, *p* = 0.02, Cohen’s *d* = 0.20), however, both mean results do not exceed the norm. Fathers experienced moderate anxiety (*M* = 10.51; *SD* = 3.87) and relatively low level of stress (*M* = 30.79; *SD* = 9.16). Mothers did not experience depression symptoms (*M* = 6.61; *SD* = 4.04), but they experienced moderate anxiety (*M* = 11.85; *SD* = 4.21) and a mild stress (*M* = 32.55; *SD* = 8.42).

**Table 2:** Means, SDs, and zero-order correlations among study variables.

S.no	Variable	*N*	*M*	*SD*	Range	1	2	3	4	5	6	7
1	Paternal postpartum bonding	131	6.63	6.68	0–33	(0.84)						
2	Paternal anxiety	131	10.51	3.87	6–28	0.34^**^	(0.88)					
3	Paternal stress	131	30.79	9.16	17–65	0.60^**^	0.41^**^	(0.88)				
4	Maternal depression symptoms	126	6.61	4.04	0–22	0.07	0.04	0.08	(0.80)			
5	Maternal postpartum bonding	129	7.95	6.64	0–30	0.45^**^	0.01	0.23^**^	0.39^**^	(0.80)		
6	Maternal anxiety	120	11.85	4.21	1–29	0.13	0.16	0.27^**^	0.70^**^	0.28^**^	(0.85)	
7	Maternal stress	125	32.55	8.42	18–57	0.30^**^	0.09	0.28^**^	0.34^**^	0.59^**^	0.33^**^	(0.83)

** *p* < 0.01, two-tailed.

Alpha coefficients are given in brackets on the diagonal.

As presented in Table 2, paternal postpartum bonding was positively related to paternal anxiety (moderate strength) and maternal stress (strong correlation). Furthermore, paternal postpartum bonding was significantly positively correlated with maternal postpartum bonding and maternal stress but there were no significant correlations between paternal postpartum bonding and maternal depression symptoms and maternal anxiety.

### Mediating Model of Paternal Anxiety on Paternal Postpartum Bonding *via* Paternal Stress

**Figure 1:**
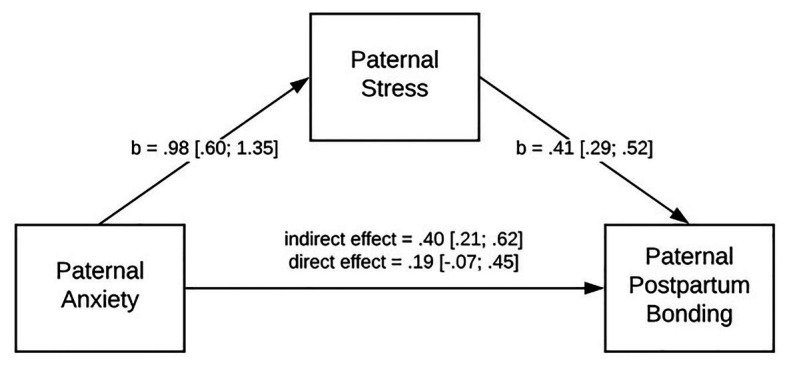
Mediating model of paternal anxiety on paternal postpartum bonding *via* paternal stress.

The rationale to create a mediating model of paternal anxiety on paternal postpartum bonding *via* paternal stress was due to findings from previous studies (e.g., Nima et al., 2013), which showed that in general stress partially mediated the effects of anxiety upon depression. Thus, we expected that parental stress could play a similar role in the relationship between parental anxiety and experiencing problematic postpartum bonding. We found that paternal stress mediates the relationship between paternal anxiety on paternal postpartum bonding. The tested model (Figure 1) proved that the path from paternal anxiety to paternal postpartum bonding (taking into account paternal stress in the model) is statistically insignificant. As we expected, paternal anxiety strengthens paternal stress (*b* = 0.98). Further, a high level of paternal stress disrupts paternal postpartum bonding (*b* = 0.41).

### Predictors of Paternal Postpartum Bonding

We have not included maternal depression symptoms and anxiety in regression analysis due to a lack of significant correlations with paternal postpartum bonding as demonstrated in the earlier phase of the analysis (Table 2). In addition, due to the fact that the relationship of paternal anxiety with paternal postpartum bonding is mediated by paternal stress, paternal anxiety was also excluded from the model.

The results, as shown in Table 3, indicate that maternal infant bonding and paternal stress are the only predictors of parental postpartum bonding across all included variables. Maternal stress is not significantly associated with paternal postpartum bonding. Moreover, the regression analysis indicated that there was no association between paternal postpartum bonding and socio-demographic factors, such as a lack of father’s presence at childbirth, being a first-time father, older age, and lower education level (Table 3).

**Table 3:** Results of regression analyses for Paternal Postpartum Bonding.

Predictor	*b*	*SE*	*p*	LLCI	ULCI
Fathers’ presence at childbirth	0.06	0.59	0.91	−1.18	1.23
Fathers’ educational level	0.58	0.33	0.08	−0.12	1.64
Fathers’ age	0.08	0.10	0.43	−0.12	0.27
Fathers’ parental experience	−0.12	0.96	0.90	−2.01	1.98
Paternal Stress	0.36	0.08	< 0.01	0.22	0.52
Maternal postpartum bonding	0.32	0.10	< 0.01	0.13	0.51
Maternal stress	−0.03	0.07	0.68	−0.16	0.11

*b*, unstandardized coefficient; SE, standard error; *p*, significant; LLCI, lower level 95% CI; and ULCI, upper level 95% CI.

## Discussion

Returning to the research questions posed at the beginning of this study, it is now possible to state that paternal postpartum bonding was positively related to paternal anxiety, maternal postpartum bonding, and maternal stress as expected. In addition, paternal anxiety strengthens paternal stress, and paternal stress mediates the relationship between paternal anxiety and paternal postpartum bonding. Contrary to expectations, this study did not find a significant correlation between postpartum bonding with maternal depression symptoms and maternal anxiety. Surprisingly, none of investigated socio-demographic variables were associated with paternal postpartum bonding. Clinically, the most relevant finding was that maternal infant bonding and paternal stress are the only predictors of parental postpartum bonding across all included variables.

With respect to the first research question, the results have shown that there is a significant positive relationship between a paternal postpartum bonding and fathers’ mental health. As expected, fathers with a high level of stress and moderate anxiety are more likely to experience problematic paternal postpartum bonding. Our results are consistent with other studies, where stress was negatively associated with the parental bonding process (Epifanio et al., 2015; McNamara et al., 2019) and anxiety as a risk factor for a problematic parent – child relationship and subsequently child development (Kim and Swain, 2007). In addition, some of other studies demonstrated that anxiety among fathers is also a risk factor for paternal depression (Matthey et al., 2003). However, in our study, paternal depression was beyond the scope of this project. The level of anxiety did not differ substantially – despite the demonstrated statistical significance – in mothers and fathers under this investigation. However, other research has showed that in comparison with mothers, fathers tend to have lower levels of anxiety in postpartum period (Matthey et al., 2003; Candelori et al., 2015). Nevertheless, at 3 months postpartum, paternal levels of anxiety were on a similar level as maternal anxiety (Figueiredo and Conde, 2011; Koh et al., 2015). Further in the results analysis, we conducted the mediation model, in which we examined the relationship between paternal stress, anxiety, and parental postpartum bonding. We found that paternal stress mediates the relationship between paternal anxiety and paternal postpartum bonding. In our study, the level of anxiety experienced by fathers strengthens the experience of stress, and a high level of paternal stress amplifies problematic paternal bonding with a newborn baby. This result is in line with the results obtained by Wee et al. (2015). In their study on expecting fathers, high levels of anxiety in early pregnancy among fathers were associated with high levels of paternal stress and depression.

This study set out with the second aim of assessing whether paternal postpartum bonding is connected with mothers’ outcomes as mental health (stress, anxiety, and depression) and maternal postpartum bonding. The current study found that paternal postpartum bonding was significantly positively correlated with maternal postpartum bonding and maternal stress as hypothesized. It is well documented that the paternal and maternal mental states are associated with each other (Dudley et al., 2001). This issue is pivotal because when both parents experience mental health difficulties, a baby’s development may be severely disrupted (Paulson et al., 2006). Further, we conducted the regression analysis, which indicated that maternal postpartum bonding and paternal stress are the only predictors of paternal postpartum bonding. In terms of these outcomes, our results are in line with other research (e.g., Epifanio et al., 2015). This differs from the findings presented by e.g., Nishigori et al. (2020), who proved that father-to-infant bonding failure was associated with maternal-to-infant bonding failure, maternal distress during pregnancy, and a father’s depressive symptoms during the postpartum period. In contrast to earlier findings, however, no significant correlation between paternal postpartum bonding and maternal depressive symptoms or anxiety was observed in our study, which means that paternal postpartum bonding did not correspond with depression or anxiety experienced by mothers. This result may be explained by the fact that mothers included in this project did not experience depression symptoms in postnatal period (they received scores below the cut-off points of 13 in EPDS indicating depressive symptoms). Current results are likely to also be related to a newborn’s condition directly after delivery (92% of them received 10 points on Apgar scale with the birth normal weight above 2.5 kg). All babies were born between 38 and 41 weeks of gestation; and what is commonly known mothers of premature birth are linked to worse mental health (Cook et al., 2018; de Paula Eduardo et al., 2019). What is interesting is that included mothers experienced moderate anxiety, which was not connected with parental postpartum bonding. A possible explanation for this might be that there is overlap between stress and anxiety, and they affect each other.

The present study also determined whether paternal postpartum bonding is associated with socio-demographic variables. Contrary to expectations, we found that a lack father’s attendance during childbirth, lack of experience with having previous child (being a first-time father), older age, and low education level were not significantly associated with paternal postpartum bonding. The findings of the current study do not support the previous research. A study conducted by Aslan et al. (2017) reported that the paternal education level and occupation were significantly related to a father’s involvement in taking care of the child. Fathers with university level education had a higher engagement in child – rearing above expected values. Although, these results differ from this data, they are consistent with others. In the same study, a father’s age, economic status, duration of marriage, number of children, attending at childbirth, or education were not correlated with participation in infant care (Aslan et al., 2017).

### Implication for Clinical Practice

The study results have important clinical implications by raising the significance for pre-screening paternal and maternal mental health in the early post-partum period to provide additional support for risk groups. This study adds to the growing body of literature showing that the early postpartum period is a vulnerable time for men. Our results have shown that fathers of a healthy newborn from full-term pregnancies are also an at-risk group of higher-level anxiety. Paternal anxiety and maternal postpartum bonding are predictors toward a problematic paternal–infant bonding. While it is essential for mothers and their babies to have a good relationship, it is also important for fathers. Growing a deep and positive paternal connection with their infants form the very beginning is important due to due to long-lasting impact of bonding on child’s psychosocial and developmental outcomes. The quality of the paternal involvement in child – bearing is crucial for a child’s cognitive, emotional, and social development during the first years and likely beyond (Ramchandani et al., 2005). What is more, studies examining paternal infant bonding found that fathers who are given more opportunities to engage in taking care of the baby can become just as nurturing parents as mothers (Kim and Swain, 2007). Thus, in research, more focus on fathers’ experiences during the antenatal and postpartum periods is needed to expand the knowledge about paternal – infant bonding and also risk factors that may cause disturbances in the bonding process. Hospital policy should pay more attention to fathers of newborns and encouraged them to engage in taking care of children. Paternal care is crucial for successful transition to fatherhood and increases self-efficiently in taking up a satisfactory parental role. Health care professional should support fathers as better parental outcomes positively impact paternal infant bonding during the first days of fatherhood. Specific interventions aimed at the promotion of early paternal postpartum bonding are needed.

### Implication for Further Research

This research has thrown up many questions that need further investigation. It would be interesting to assess the trajectory of bonding over time, because studies reported that adjustment to fatherhood was connected to various variables at different times in the perinatal and postnatal periods (Matthey et al., 2000). Thus, a prospective cohort study with a longitudinal study design would be valuable. In addition, more research is required to determine how paternal/maternal mental health and socio-demographic factors affect paternal bonding is a cross-national study.

### Limitation

The reader should bear in mind the limitations of this study to make appropriate interpretation of the study results. The generalization of this data is problematic because the study was conducted in one unit including a small sample size. This study is unable to encompass the entire issue due to the fact that statistical analysis applies to fathers of newborns from single pregnancies, healthy babies born full-time with normal birth weight. It is important to acknowledge that using self-reports increases the risk of bias in comparison with clinically observed outcomes with unblinded designs. However, due to a nature of undertaken subject, cross-sectional study seems to be the most efficient and sensitive aspect of data collection. The results of the study should be interpreted with caution because the measurement tools (PBQ and PSS questionnaires) may require further validation studies.

### Conclusion

This study has identified that paternal postpartum bonding was positively related to paternal anxiety and maternal stress, maternal postpartum bonding, and maternal stress. The second major finding was that maternal infant bonding and paternal stress are the only predictors of parental postpartum bonding across all included variables. The current findings add to a growing body of literature on paternal postpartum bonding. The key strength of this study was targeting fathers who belong to an untested group in this topic. While it is essential for mothers and their babies to have a good relationship, it is also important for fathers to grow a deep connection with their babies form the very beginnings, due to long-lasting impact of bonding on child’s psychosocial and mental development (Ramchandani et al., 2005). The results highlight the need for interventions concentrating on paternal postpartum bonding and parental mental health in order to help fathers grow a positive bond with their child.

## Data Availability Statement

The data that support the findings of this study are available on request from the corresponding author. The data are not publicly available due to their containing information that could compromise the privacy of research participants.

## Ethics Statement

The studies involving human participants were reviewed and approved by the Research Ethics Board at the University of Gdansk (no 7/2019, date of approval: April 29, 2019). The patients/participants provided their written informed consent to participate in this study.

## Author Contributions

ŁB, MB, and KL: conceptualization. PJ: formal analysis. ŁB and MB: investigation, project administration, and supervision. PJ, ŁB, MB, and KL: methodology. ŁB, KL, and PJ: writing – original draft preparation. All authors contributed to the article and approved the submitted version.

### Conflict of Interest

The authors declare that the research was conducted in the absence of any commercial or financial relationships that could be construed as a potential conflict of interest.

## Footnotes

^1^World Health Organization (2020). Available at: https://www.who.int/substance_abuse/research_tools/translation/en/ (Accessed June, 1, 2020).
