# Supplementary material for: Paternal Postpartum Bonding and Its Predictors in the Early Postpartum Period: Cross-Sectional Study in a Polish Cohort
Source: Front Psychol. 2021 Apr 9;12:628650. doi: 10.3389/fpsyg.2021.628650 (PMC8062924; doi:10.3389/fpsyg.2021.628650)
Supplement: Supplementary file 1 [file Table_1.DOCX]

Supplementary Material

# Supplementary Table S1. EFA standardized factor loadings for PBQ items using Polish sample.

| **Item** | **Item content** | **Original factor** | **F1** | **F2** | **F3** | **F4** |
| --- | --- | --- | --- | --- | --- | --- |
| PBQ6 | The baby does not seem to be mine | F1 | **.93** |  |  |  |
| PBQ17 | I wish my baby would somehow go away | F1 | **.90** |  |  |  |
| PBQ23 | I feel the only solutions is for someone else to look after my baby | F2 | **.85** |  |  |  |
| PBQ5 | I regret having this baby | F2 | **.65** | .61 |  |  |
| PBQ19 | My baby makes me feel anxious | F3 | **.60** |  | .41 |  |
| PBQ9 | I feel happy when my baby smiles or laughs [r] | F1 |  | **.86** |  |  |
| PBQ8 | I love my baby to bits (r) | F1 |  | **.81** |  |  |
| PBQ1 | I feel close to my baby (r) | F1 |  | **.69** |  | .42 |
| PBQ16 | My baby is the most beautiful baby in the world (r) | F1 | .50 | **.59** |  |  |
| PBQ3 | I feel distant from my baby | F2 |  | **.51** | .32 |  |
| PBQ11 | I enjoy playing with my baby (r) | F2 |  | **.50** | .47 | .34 |
| PBQ22 | I feel confident when caring for my baby (r) | F3 | .36 | **.49** |  |  |
| PBQ21 | My baby annoys me | F2 |  |  | **.78** |  |
| PBQ10 | My baby irritates me | F1 |  |  | **.77** |  |
| PBQ7 | My baby winds me up | F1 |  |  | **.71** |  |
| PBQ14 | I feel angry with my baby | F2 | .36 |  | **.61** |  |
| PBQ2 | I wish the old days when I had no baby would come back | F1 |  | .40 | **.57** |  |
| PBQ13 | I feel trapped as a parent | F1 |  |  | **.53** |  |
| PBQ12 | My baby cries too much | F1 |  |  | **.45** |  |
| PBQ18 | I have done harmful things to my baby | F4 |  |  |  | **.84** |
| PBQ24 | I feel like hurting my baby | F4 |  |  |  | **.81** |
| PBQ4 | I love to cuddle my baby (r) | F2 |  | .42 | .30 | **.66** |

*Note.* For clarity, factor loadings below 0.3 have been hidden.

*Abbreviations*: EFA = exploratory factor analysis; PBQ = Postpartum Bonding Questionnaire

**
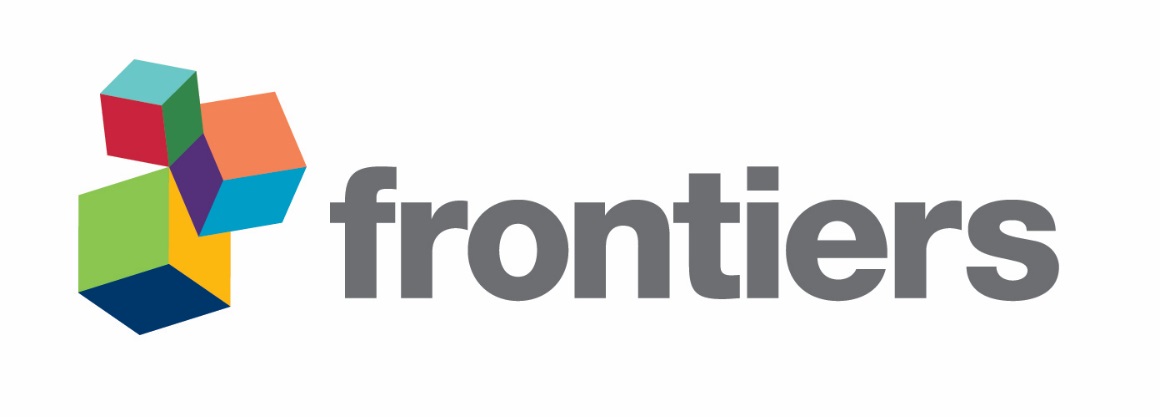
**
